# Supplementary material for: Cytokines and chemokines profile in encephalitis patients: A meta-analysis
Source: PLoS One. 2022 Sep 1;17(9):e0273920. doi: 10.1371/journal.pone.0273920 (PMC9436077; doi:10.1371/journal.pone.0273920)
Supplement: S1 Table — (PDF) [file pone.0273920.s002.pdf]

Search results for cytokines in encephalitis

	Database	Search string
1	PubMed	((("encephalitis"[Title/Abstract]) OR ("tick borne encephalitis"[Title/Abstract]) OR ("hhv 6 encephalitis"[Title/Abstract]) OR ("hhv6 encephalitis"[Title/Abstract]) OR ("tickborne encephalitis"[Title/Abstract]) OR ("hhv 6b encephalitis"[Title/Abstract]) OR ("hhv 6a encephalitis"[Title/Abstract]) OR ("hhv 7 encephalitis"[Title/Abstract]) OR ("japanese encephalitis"[Title/Abstract]) OR ("acute disseminated encephalitis"[Title/Abstract]) OR ("acute disseminated encephalomyelitis"[Title/Abstract]) OR ("adem"[Title/Abstract]) OR ("acute demyelinating encephalitis"[Title/Abstract]) OR ("acute demyelinating encephalomyelitis"[Title/Abstract]) OR ("antinmdarencephalitis"[Title/Abstract]) OR ("anti nmdar encephalitis"[Title/Abstract]) OR ("anti nmda receptor encephalitis"[Title/Abstract]) OR ("autoimmune encephalitis"[Title/Abstract]) OR ("auto immune encephalitis"[Title/Abstract]) OR ("auto immune encephalomyelitis"[Title/Abstract]) OR ("enteroviral encephalitis"[Title/Abstract]) OR ("enteroviral encephalomyelitis"[Title/Abstract]) OR ("viral encephalitides"[Title/Abstract]) OR ("viral encephalitis"[Title/Abstract]) OR ("bacterial encephalitis"[Title/Abstract]) OR ("fungal encephalitis"[Title/Abstract]) OR ("meningeal encephalitis"[Title/Abstract]) OR ("zika virus associated encephalitis"[Title/Abstract]) OR ("acute encephalitis syndrome"[Title/Abstract]) OR ("rasmussen encephalitis"[Title/Abstract]) OR ("primary encephalitis"[Title/Abstract]) OR ("secondary encephalitis"[Title/Abstract]) OR ("arbovirus encephalitis"[Title/Abstract]) OR ("virus encephalitis"[Title/Abstract]) OR ("rabies encephalitis"[Title/Abstract]))) AND ((("il 1"[All Fields]) OR ("il1"[All Fields]) OR ("cytokine"[All Fields]) OR ("cytokines"[All Fields]) OR ("chemokine"[All Fields]) OR ("chemokines"[All Fields]) OR ("lymphokine"[All Fields]) OR ("lymphokines"[All Fields]) OR ("il1b"[All Fields]) OR ("il 1b"[All Fields]) OR ("il2"[All Fields]) OR ("il 2"[All Fields]) OR ("il3"[All Fields]) OR ("il 3"[All Fields]) OR ("il4"[All Fields]) OR ("il 4"[All Fields]) OR ("il5"[All Fields]) OR ("il 5"[All Fields]) OR ("il6"[All Fields]) OR ("il 6"[All Fields]) OR ("il7"[All Fields]) OR ("il 7"[All Fields]) OR ("il8"[All Fields]) OR ("il 8"[All Fields]) OR ("il9"[All Fields]) OR ("il 9"[All Fields]) OR ("il10"[All Fields]) OR ("il 10"[All Fields]) OR ("il12"[All Fields]) OR ("il 12"[All Fields]) OR ("il13"[All Fields]) OR ("il 13"[All Fields]) OR ("il17"[All Fields]) OR ("il 17"[All Fields]) OR ("il17a"[All Fields]) OR ("il 17a"[All Fields]) OR ("cxcl1"[All Fields]) OR ("cxcl 1"[All Fields]) OR ("cxcl10"[All Fields]) OR ("cxcl 10"[All Fields]) OR ("gcsf"[All Fields]) OR ("g csf"[All Fields]) OR ("granulocyte colony stimulating factor"[All Fields]) OR ("mcp"[All Fields]) OR ("ccl2"[All Fields]) OR ("ccl 2"[All Fields]) OR ("hgf"[All Fields]) OR ("hepatocyte growth factor"[All Fields]) OR ("ifn alpha"[All Fields]) OR ("ifn a"[All Fields]) OR ("ifnalpha"[All Fields]) OR ("interferon alpha"[All Fields]) OR ("tnf a"[All Fields]) OR ("tnf alpha"[All Fields]) OR ("tumor necrosis factor alpha"[All Fields]) OR ("cxcl13"[All Fields]) OR ("cxcl 13"[All Fields]) OR ("cxcl 10"[All Fields]) OR ("cxcl10"[All Fields]) OR ("ip10"[All Fields]) OR ("ip 10"[All Fields]) OR ("il 21"[All Fields]) OR ("il21"[All Fields]) OR ("mig"[All Fields]) OR ("cxcl9"[All Fields]) OR ("cxcl 9"[All Fields]) OR ("mcp 1"[All Fields]) OR ("mcp1"[All Fields]) OR ("ccl2"[All Fields]) OR ("ccl 2"[All Fields]) OR ("ccl5"[All Fields]) OR ("ccl 5"[All Fields]) OR ("cxcr 3"[All Fields]) OR ("cxcr3"[All Fields])))
2	WoS	(TI=(("encephalitis") OR ("tick borne encephalitis") OR ("hhv 6 encephalitis") OR ("hhv6 encephalitis") OR ("tickborne encephalitis") OR ("hhv 6b encephalitis") OR ("hhv 6a encephalitis") OR ("hhv 7 encephalitis") OR ("japanese encephalitis") OR ("acute disseminated encephalitis") OR ("acute disseminated encephalomyelitis") OR ("adem") OR ("acute demyelinating encephalitis") OR ("acute demyelinating encephalomyelitis") OR ("antinmdarencephalitis") OR ("anti nmdar encephalitis") OR ("anti nmda receptor encephalitis") OR ("autoimmune encephalitis") OR ("auto immune encephalitis") OR ("auto immune encephalomyelitis") OR ("enteroviral encephalitis") OR ("enteroviral encephalomyelitis") OR ("viral encephalitides") OR ("viral encephalitis") OR ("bacterial

		encephalitis") OR ("fungal encephalitis") OR ("meningeal encephalitis") OR ("zika virus associated encephalitis") OR ("acute encephalitis syndrome") OR ("rasmussen encephalitis") OR ("primary encephalitis") OR ("secondary encephalitis") OR ("arbovirus encephalitis") OR ("virus encephalitis") OR ("rabies encephalitis")) OR AB=(("encephalitis") OR ("tick borne encephalitis") OR ("hhv 6 encephalitis") OR ("hhv6 encephalitis") OR ("tickborne encephalitis") OR ("hhv 6b encephalitis") OR ("hhv 6a encephalitis") OR ("hhv 7 encephalitis") OR ("japanese encephalitis") OR ("acute disseminated encephalitis") OR ("acute disseminated encephalomyelitis") OR ("adem") OR ("acute demyelinating encephalitis") OR ("acute demyelinating encephalomyelitis") OR ("antinmdarencephalitis") OR ("anti nmdar encephalitis") OR ("anti nmda receptor encephalitis") OR ("autoimmune encephalitis") OR ("auto immune encephalitis") OR ("auto immune encephalomyelitis") OR ("enteroviral encephalitis") OR ("enteroviral encephalomyelitis") OR ("viral encephalitides") OR ("viral encephalitis") OR ("bacterial encephalitis") OR ("fungal encephalitis") OR ("meningeal encephalitis") OR ("zika virus associated encephalitis") OR ("acute encephalitis syndrome") OR ("rasmussen encephalitis") OR ("primary encephalitis") OR ("secondary encephalitis") OR ("arbovirus encephalitis") OR ("virus encephalitis") OR ("rabies encephalitis"))) AND (TI=(("il 1") OR ("il1") OR ("cytokine") OR ("cytokines") OR ("chemokine") OR ("chemokines") OR ("lymphokine") OR ("lymphokines") OR ("il1b") OR ("il 1b") OR ("il2") OR ("il 2") OR ("il3") OR ("il 3") OR ("il4") OR ("il 4") OR ("il5") OR ("il 5") OR ("il6") OR ("il 6") OR ("il7") OR ("il 7") OR ("il8") OR ("il 8") OR ("il9") OR ("il 9") OR ("il10") OR ("il 10") OR ("il12") OR ("il 12") OR ("il13") OR ("il 13") OR ("il17") OR ("il 17") OR ("il17a") OR ("il 17a") OR ("cxcl1") OR ("cxcl 1") OR ("cxcl10") OR ("cxcl 10") OR ("gcsf") OR ("g csf") OR ("granulocyte colony stimulating factor") OR ("mcp") OR ("ccl2") OR ("ccl 2") OR ("hgf") OR ("hepatocyte growth factor") OR ("ifn alpha") OR ("ifn a") OR ("ifnalpha") OR ("interferon alpha") OR ("tnf a") OR ("tnf alpha") OR ("tumor necrosis factor alpha") OR ("cxcl13") OR ("cxcl 13") OR ("cxcl 10") OR ("cxcl10") OR ("ip10") OR ("ip 10") OR ("il 21")) OR AB=(("il 1") OR ("il1") OR ("cytokine") OR ("cytokines") OR ("chemokine") OR ("chemokines") OR ("lymphokine") OR ("lymphokines") OR ("il1b") OR ("il 1b") OR ("il2") OR ("il 2") OR ("il3") OR ("il 3") OR ("il4") OR ("il 4") OR ("il5") OR ("il 5") OR ("il6") OR ("il 6") OR ("il7") OR ("il 7") OR ("il8") OR ("il 8") OR ("il9") OR ("il 9") OR ("il10") OR ("il 10") OR ("il12") OR ("il 12") OR ("il13") OR ("il 13") OR ("il17") OR ("il 17") OR ("il17a") OR ("il 17a") OR ("cxcl1") OR ("cxcl 1") OR ("cxcl10") OR ("cxcl 10") OR ("gcsf") OR ("g csf") OR ("granulocyte colony stimulating factor") OR ("mcp") OR ("ccl2") OR ("ccl 2") OR ("hgf") OR ("hepatocyte growth factor") OR ("ifn alpha") OR ("ifn a") OR ("ifnalpha") OR ("interferon alpha") OR ("tnf a") OR ("tnf alpha") OR ("tumor necrosis factor alpha") OR ("cxcl13") OR ("cxcl 13") OR ("cxcl 10") OR ("cxcl10") OR ("ip10") OR ("ip 10") OR ("il 21") OR ("il21") OR ("mig") OR ("cxcl9") OR ("cxcl 9") OR ("mcp 1") OR ("mcp1") OR ("ccl2") OR ("ccl 2") OR ("ccl5") OR ("ccl 5") OR ("cxcr 3") OR ("cxcr3")))
3	Embase	('encephalitis':ti,ab OR 'tick borne encephalitis':ti,ab OR 'hhv 6 encephalitis':ti,ab OR 'hhv6 encephalitis':ti,ab OR 'tickborne encephalitis':ti,ab OR 'hhv 6b encephalitis':ti,ab OR 'hhv 6a encephalitis':ti,ab OR 'hhv 7 encephalitis':ti,ab OR 'japanese encephalitis':ti,ab OR 'acute disseminated encephalitis':ti,ab OR 'acute disseminated encephalomyelitis':ti,ab OR 'adem':ti,ab OR 'acute demyelinating encephalitis':ti,ab OR 'acute demyelinating encephalomyelitis':ti,ab OR 'antinmdarencephalitis':ti,ab OR 'anti nmdar encephalitis':ti,ab OR 'anti nmda receptor encephalitis':ti,ab OR 'autoimmune encephalitis':ti,ab OR 'auto immune encephalitis':ti,ab OR 'auto immune encephalomyelitis':ti,ab OR 'enteroviral encephalitis':ti,ab OR 'enteroviral encephalomyelitis':ti,ab OR 'viral encephalitides':ti,ab OR 'viral encephalitis':ti,ab OR 'bacterial encephalitis':ti,ab OR 'fungal encephalitis':ti,ab OR 'meningeal encephalitis':ti,ab OR 'zika virus associated encephalitis':ti,ab OR 'acute encephalitis syndrome':ti,ab OR 'rasmussen encephalitis':ti,ab OR 'primary encephalitis':ti,ab OR 'secondary encephalitis':ti,ab OR 'arbovirus encephalitis':ti,ab OR 'virus

		encephalitis':ti,ab OR 'rabies encephalitis':ti,ab) AND ('il 1':ti,ab OR 'il1':ti,ab OR 'cytokine':ti,ab OR 'cytokines':ti,ab OR 'chemokine':ti,ab OR 'chemokines':ti,ab OR 'lymphokine':ti,ab OR 'lymphokines':ti,ab OR 'il1b':ti,ab OR 'il 1b':ti,ab OR 'il2':ti,ab OR 'il 2':ti,ab OR 'il3':ti,ab OR 'il 3':ti,ab OR 'il4':ti,ab OR 'il 4':ti,ab OR 'il5':ti,ab OR 'il 5':ti,ab OR 'il6':ti,ab OR 'il 6':ti,ab OR 'il7':ti,ab OR 'il 7':ti,ab OR 'il8':ti,ab OR 'il 8':ti,ab OR 'il9':ti,ab OR 'il 9':ti,ab OR 'il10':ti,ab OR 'il 10':ti,ab OR 'il12':ti,ab OR 'il 12':ti,ab OR 'il13':ti,ab OR 'il 13':ti,ab OR 'il17':ti,ab OR 'il 17':ti,ab OR 'il17a':ti,ab OR 'il 17a':ti,ab OR 'cxcl1':ti,ab OR 'cxcl 1':ti,ab OR 'gcsf':ti,ab OR 'g csf':ti,ab OR 'granulocyte colony stimulating factor':ti,ab OR 'mcp':ti,ab OR 'ccl2':ti,ab OR 'ccl 2':ti,ab OR 'hgf':ti,ab OR 'hepatocyte growth factor':ti,ab OR 'ifn alpha':ti,ab OR 'ifn a':ti,ab OR 'ifnalpha':ti,ab OR 'interferon alpha':ti,ab OR 'tnf a':ti,ab OR 'tnf alpha':ti,ab OR 'tumor necrosis factor alpha':ti,ab OR 'cxcl13':ti,ab OR 'cxcl 13':ti,ab OR 'cxcl 10':ti,ab OR 'cxcl10':ti,ab OR 'ip10':ti,ab OR 'ip 10':ti,ab OR 'il 21':ti,ab)
4	Scopus	TITLE-ABS (*"il 1"* OR *"il1"* OR *"cytokine"* OR *"cytokines"* OR *"chemokine"* OR *"chemokines"* OR *"lymphokine"* OR *"lymphokines"* OR *"il1b"* OR *"il 1b"* OR *"il2"* OR *"il 2"* OR *"il3"* OR *"il 3"* OR *"il4"* OR *"il 4"* OR *"il5"* OR *"il 5"* OR *"il6"* OR *"il 6"* OR *"il7"* OR *"il 7"* OR *"il8"* OR *"il 8"* OR *"il9"* OR *"il 9"* OR *"il10"* OR *"il 10"* OR *"il12"* OR *"il 12"* OR *"il13"* OR *"il 13"* OR *"il17"* OR *"il 17"* OR *"il17a"* OR *"il 17a"* OR *"cxcl1"* OR *"cxcl 1"* OR *"cxcl10"* OR *"cxcl 10"* OR *"gcsf"* OR *"g csf"* OR *"granulocyte colony stimulating factor"* OR *"mcp"* OR *"ccl2"* OR *"ccl 2"* OR *"hgf"* OR *"hepatocyte growth factor"* OR *"ifn alpha"* OR *"ifn a"* OR *"ifnalpha"* OR *"interferon alpha"* OR *"tnf a"* OR *"tnf alpha"* OR *"tumor necrosis factor alpha"* OR *"cxcl13"* OR *"cxcl 13"* OR *"cxcl 10"* OR *"cxcl10"* OR *"ip10"* OR *"ip 10"* OR *"il 21"* OR *"il21"* OR *"mig"* OR *"cxcl9"* OR *"cxcl 9"* OR *"mcp 1"* OR *"mcp1"* OR *"ccl2"* OR *"ccl 2"* OR *"ccl5"* OR *"ccl 5"* OR *"cxcr 3"* OR *"cxcr3"*) AND TITLE-ABS (*"encephalitis"* OR *"tick borne encephalitis"* OR *"hhv 6 encephalitis"* OR *"hhv6 encephalitis"* OR *"tickborne encephalitis"* OR *"hhv 6b encephalitis"* OR *"hhv 6a encephalitis"* OR *"hhv 7 encephalitis"* OR *"japanese encephalitis"* OR *"acute disseminated encephalitis"* OR *"acute disseminated encephalomyelitis"* OR *"adem"* OR *"acute demyelinating encephalitis"* OR *"acute demyelinating encephalomyelitis"* OR *"antinmdarencephalitis"* OR *"anti nmdar encephalitis"* OR *"anti nmda receptor encephalitis"* OR *"autoimmune encephalitis"* OR *"auto immune encephalitis"* OR *"auto immune encephalomyelitis"* OR *"enteroviral encephalitis"* OR *"enteroviral encephalomyelitis"* OR *"viral encephalitides"* OR *"viral encephalitis"* OR *"bacterial encephalitis"* OR *"fungal encephalitis"* OR *"meningeal encephalitis"* OR *"zika virus associated encephalitis"* OR *"acute encephalitis syndrome"* OR *"rasmussen encephalitis"* OR *"primary encephalitis"* OR *"secondary encephalitis"* OR *"arbovirus encephalitis"* OR *"virus encephalitis"* OR *"rabies encephalitis"*)
