# Supplementary material for: Cytokines and chemokines profile in encephalitis patients: A meta-analysis
Source: PLoS One. 2022 Sep 1;17(9):e0273920. doi: 10.1371/journal.pone.0273920 (PMC9436077; doi:10.1371/journal.pone.0273920)

IL-2 CSF

Funnel plot

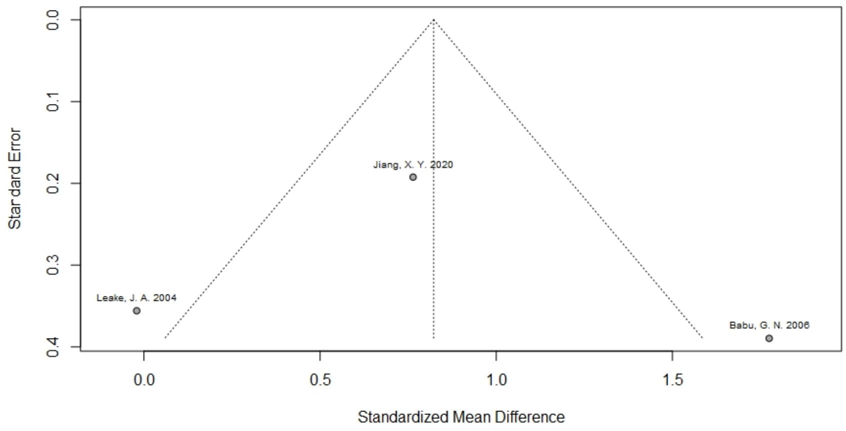

Drapery plot

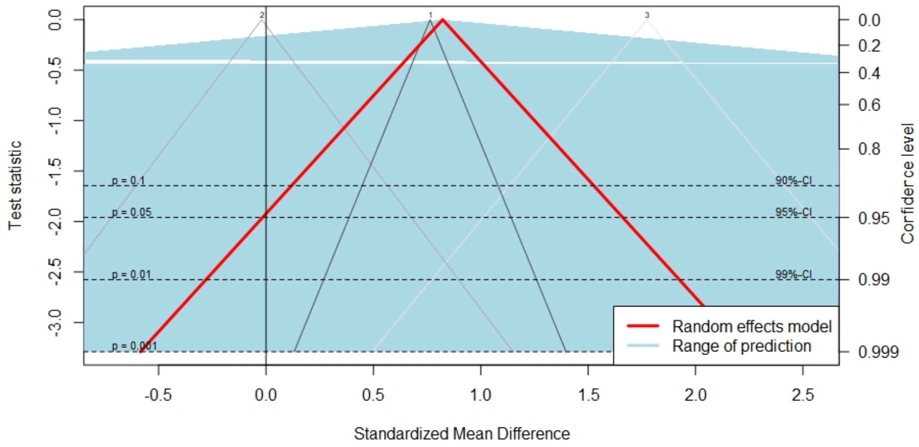

Leave-one-out analysis

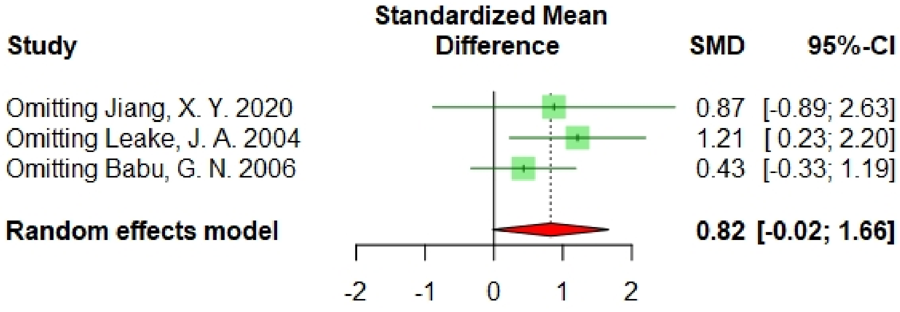

IL-4 CSF

Funnel plot

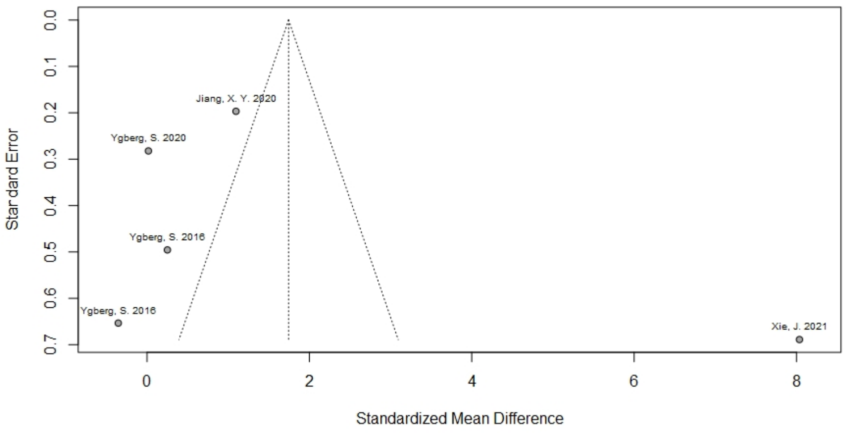

Drapery plot

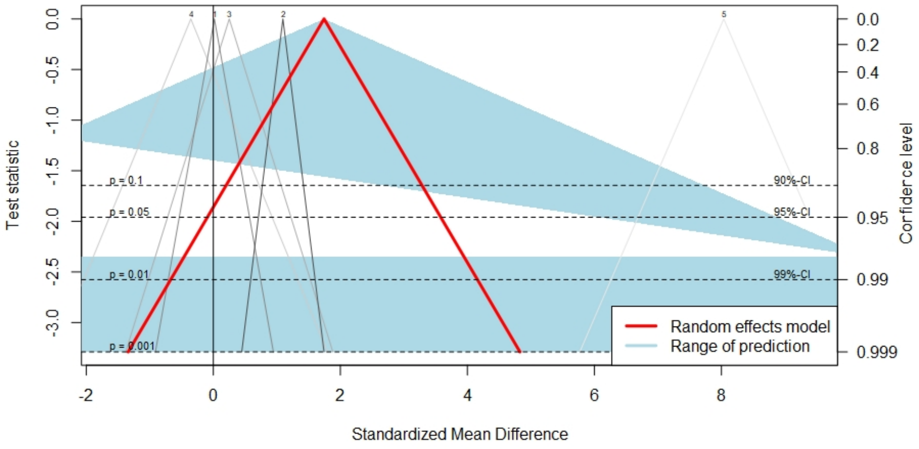

Leave-one-out analysis

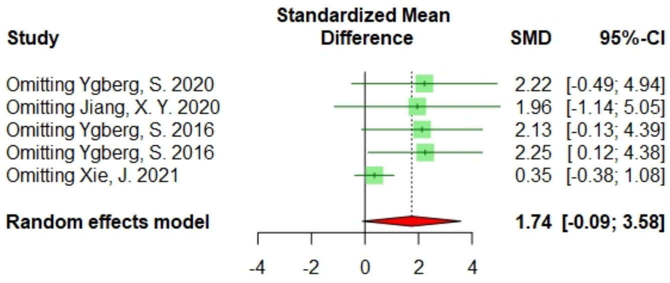

IL-6 CSF

Funnel plot

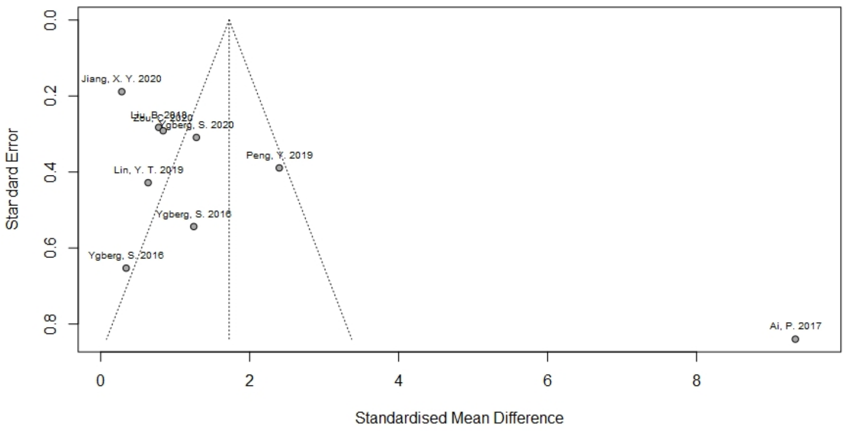

Drapery plot

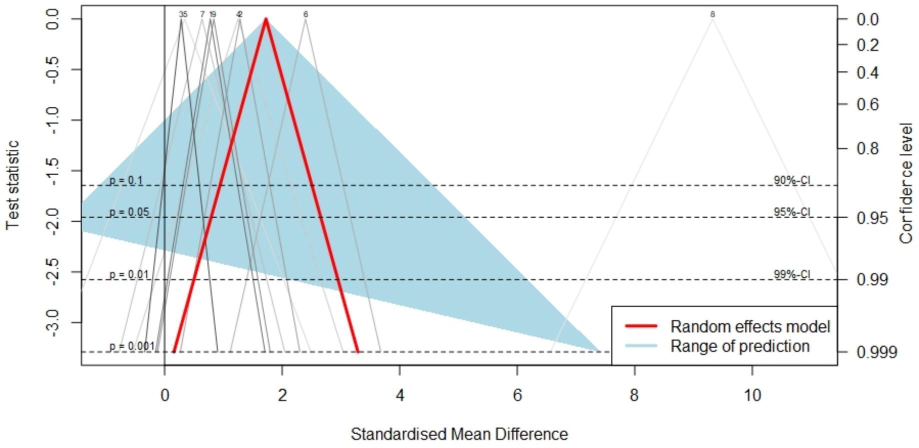

Leave-one-out analysis

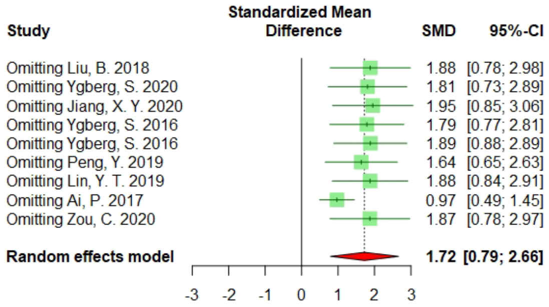

IL-6 serum

Funnel plot

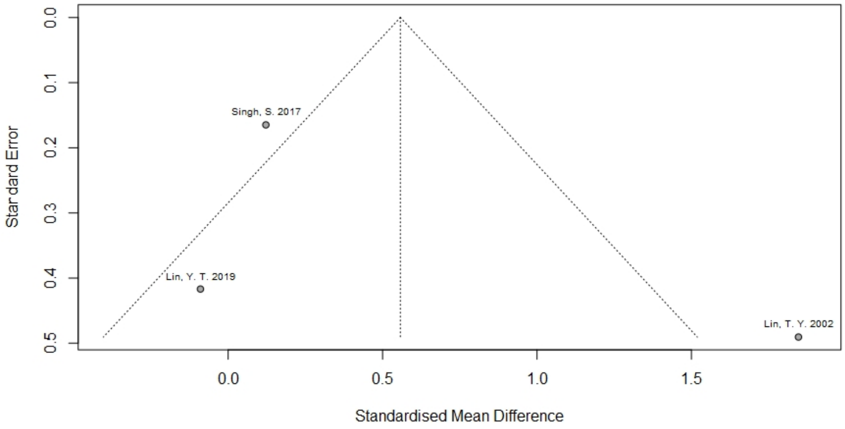

Drapery plot

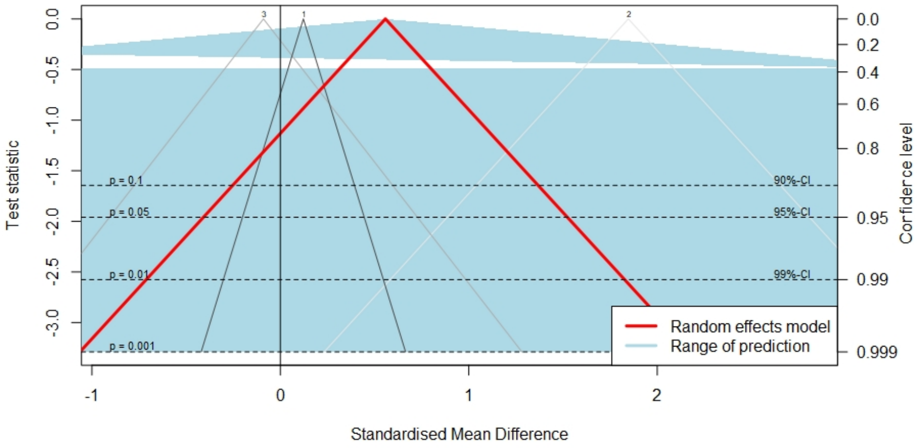

Leave-one-out analysis

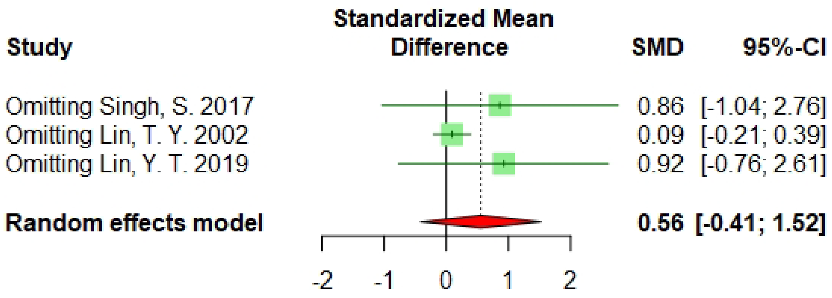

IL-8 CSF

Funnel plot

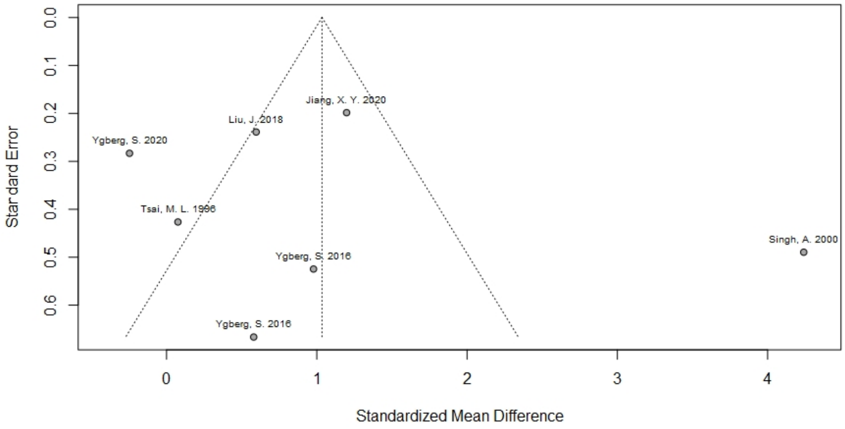

Drapery plot

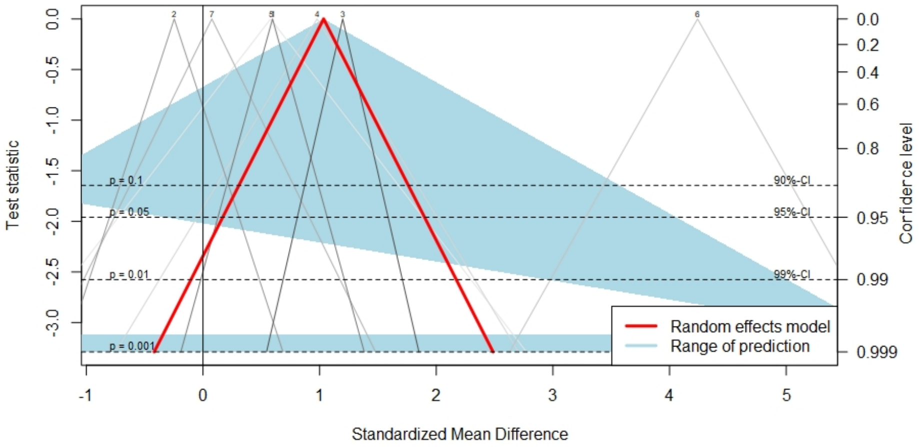

Leave-one-out analysis

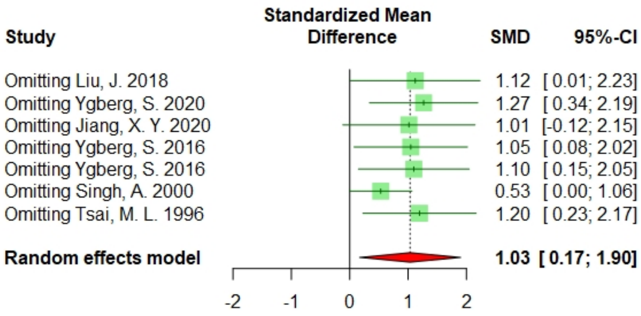

IL-10 CSF

Funnel plot

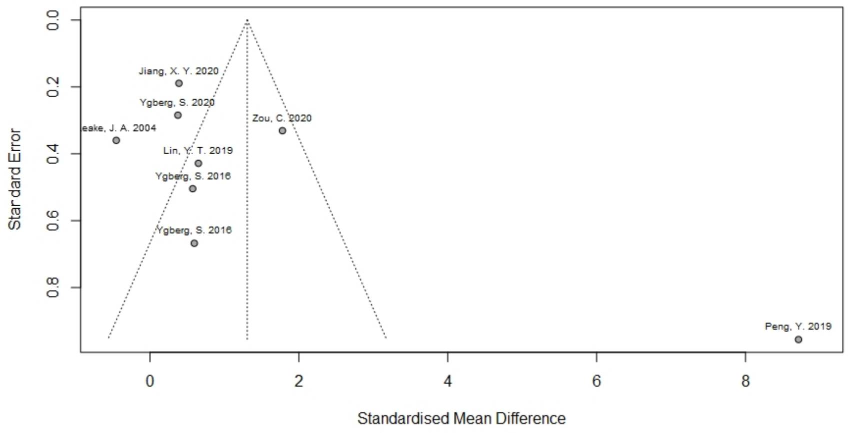

Drapery plot

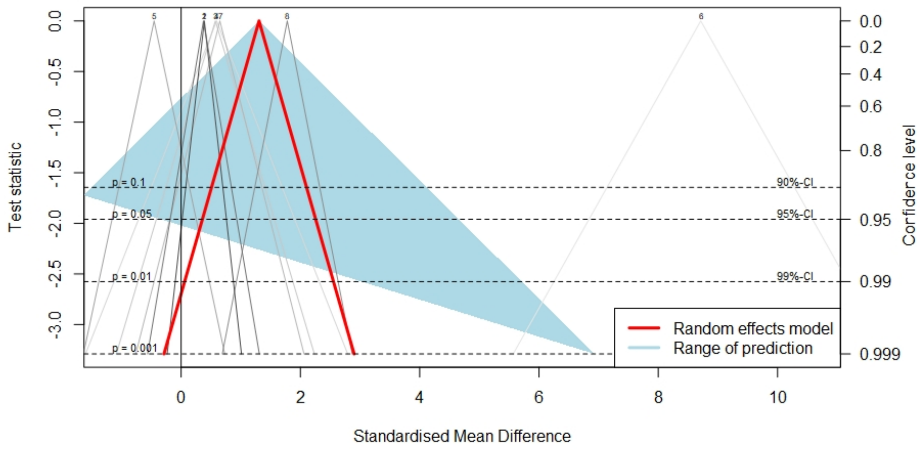

Leave-one-out analysis

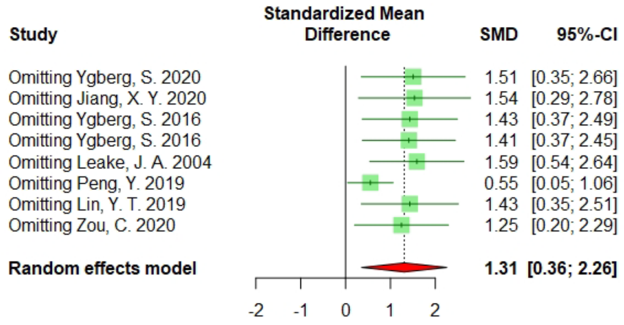

IL-10 serum

Funnel plot

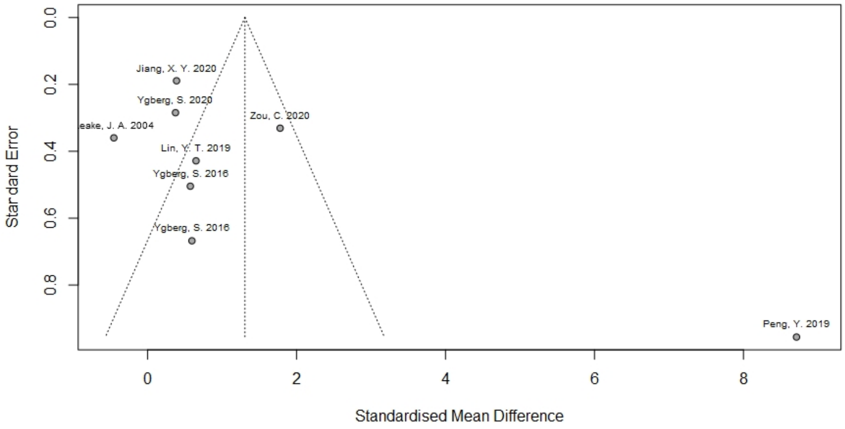

Drapery plot

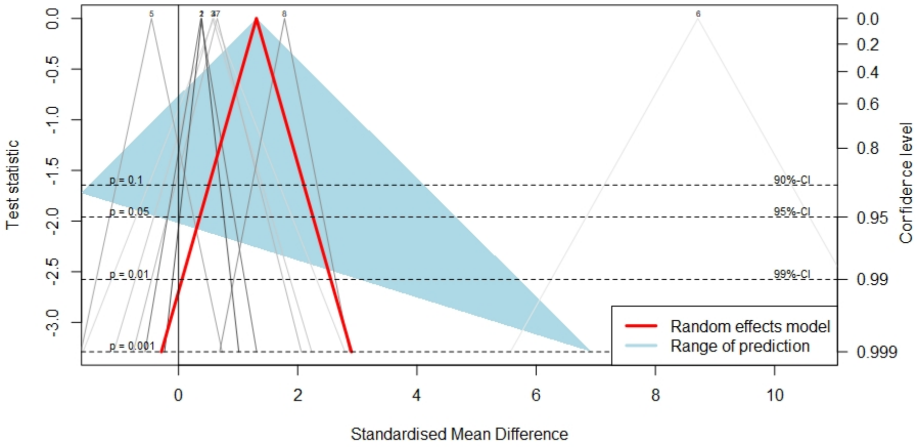

Leave-one-out analysis

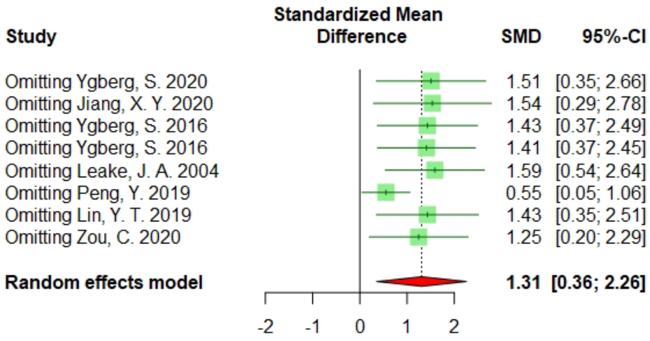

IL-17

Funnel plot

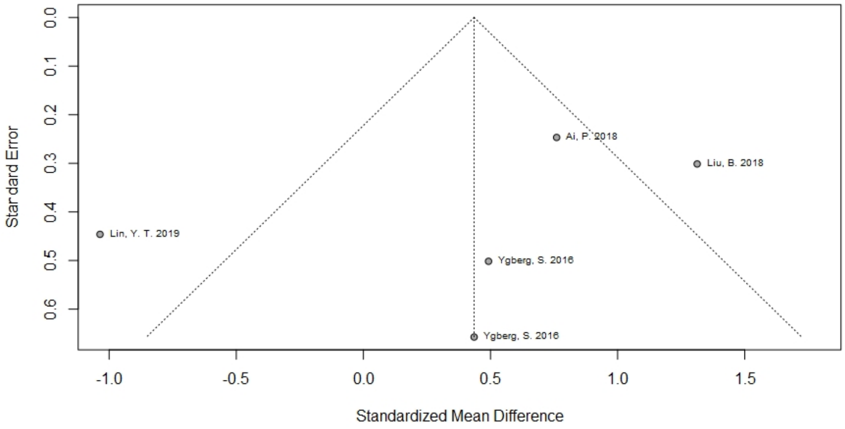

Drapery plot

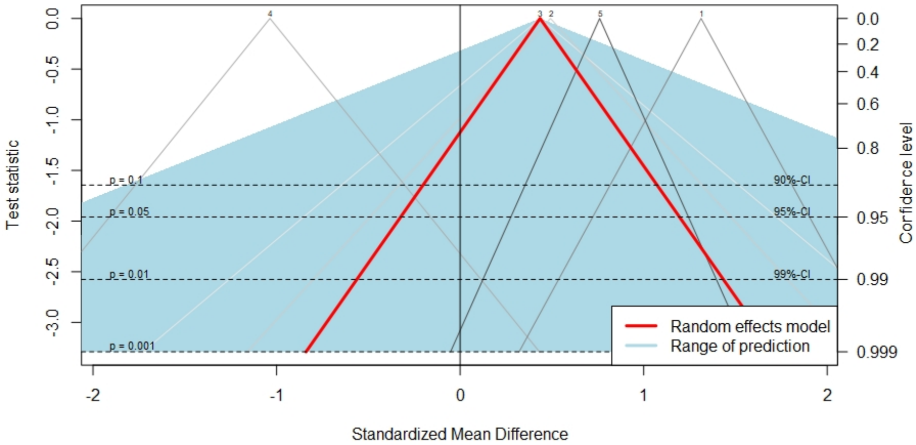

Leave-one-out analysis

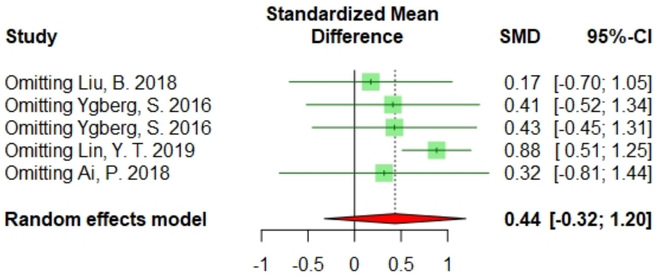

CCL2

Funnel plot

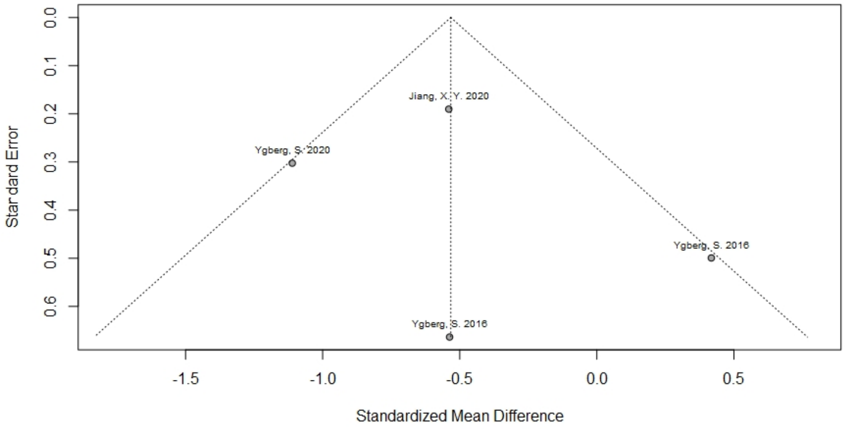

Drapery plot

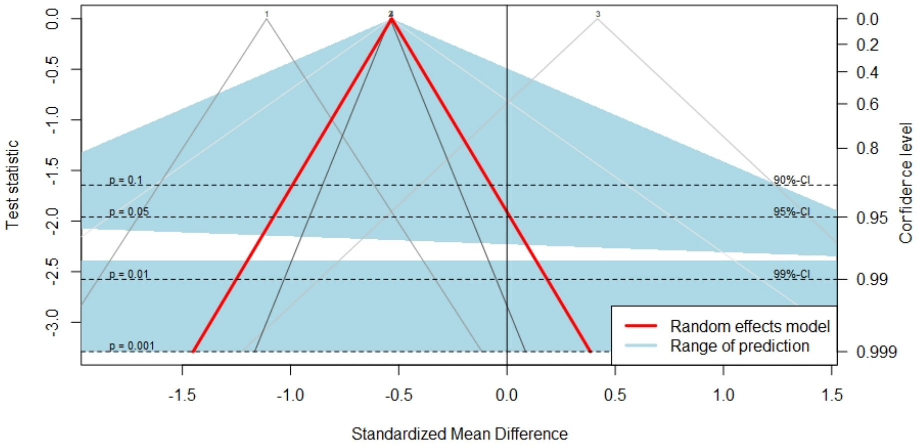

Leave-one-out analysis

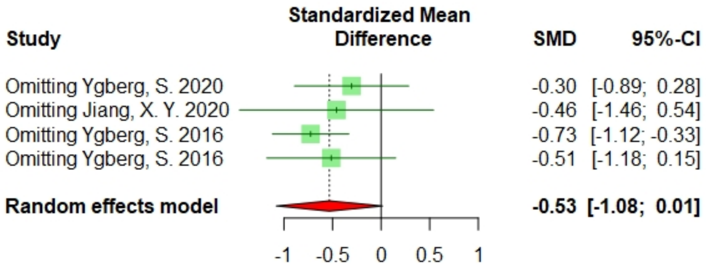

CXCL9

Funnel plot

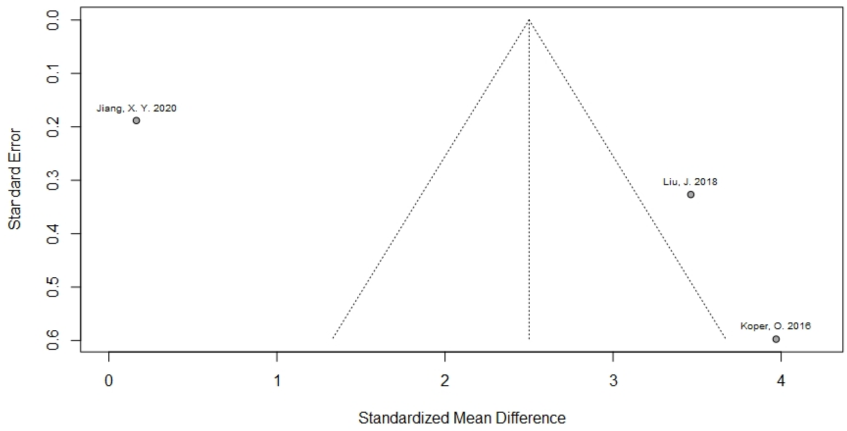

Drapery plot

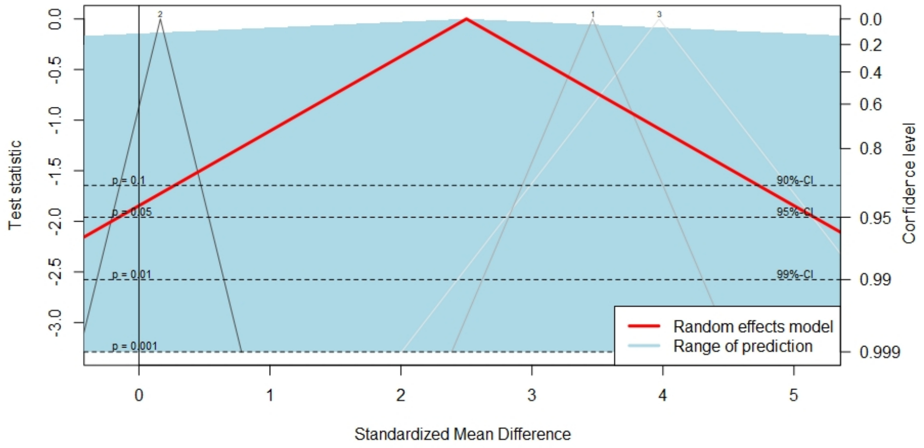

Leave-one-out analysis

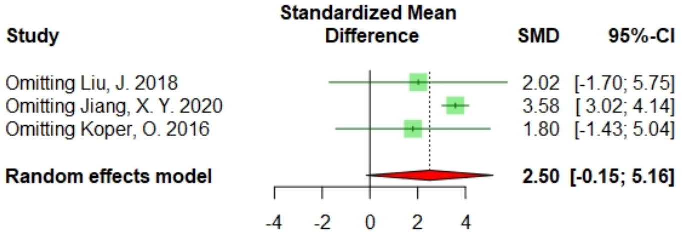

CXCL10

Funnel plot

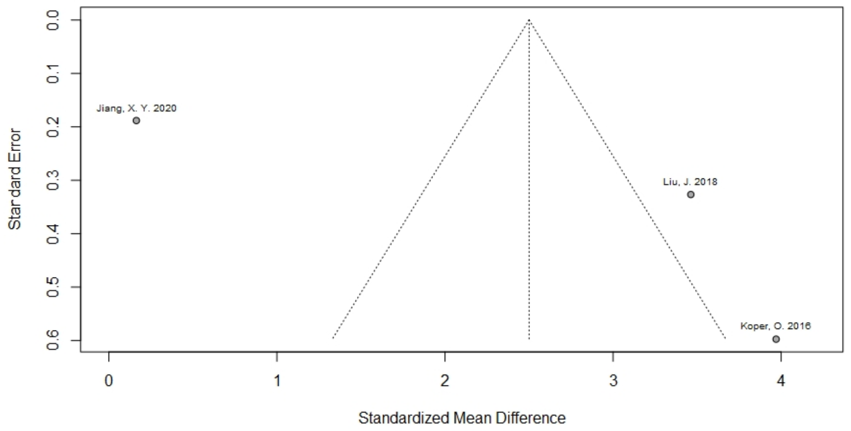

Drapery plot

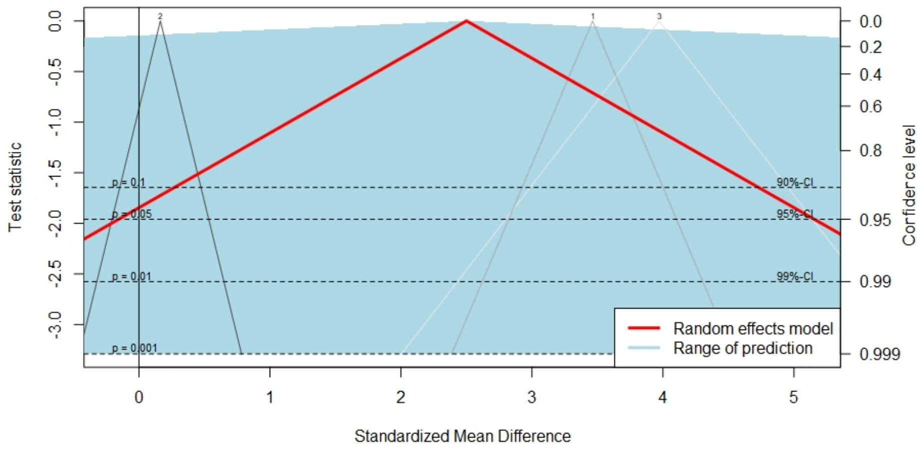

Leave-one-out analysis

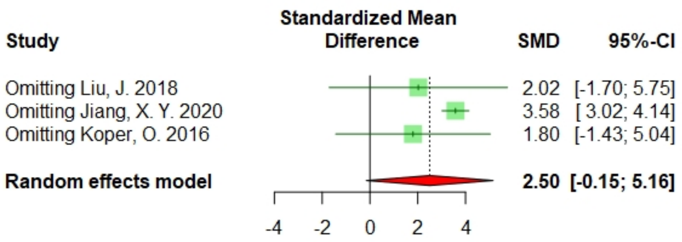

CXCL10 CSF

Funnel plot

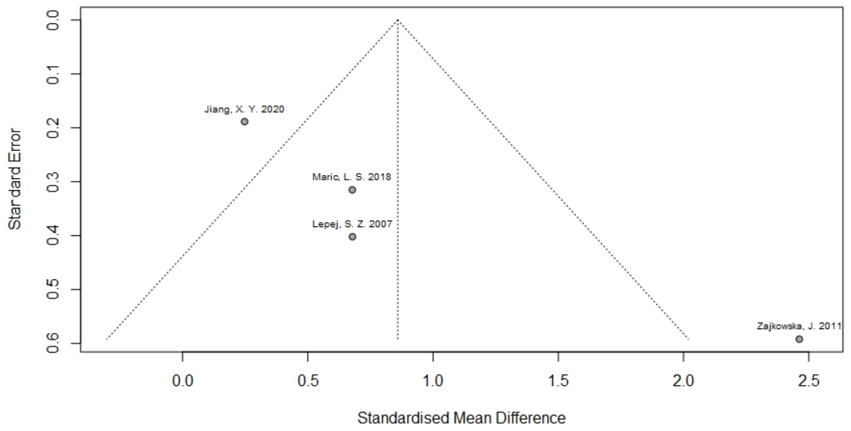

Drapery plot

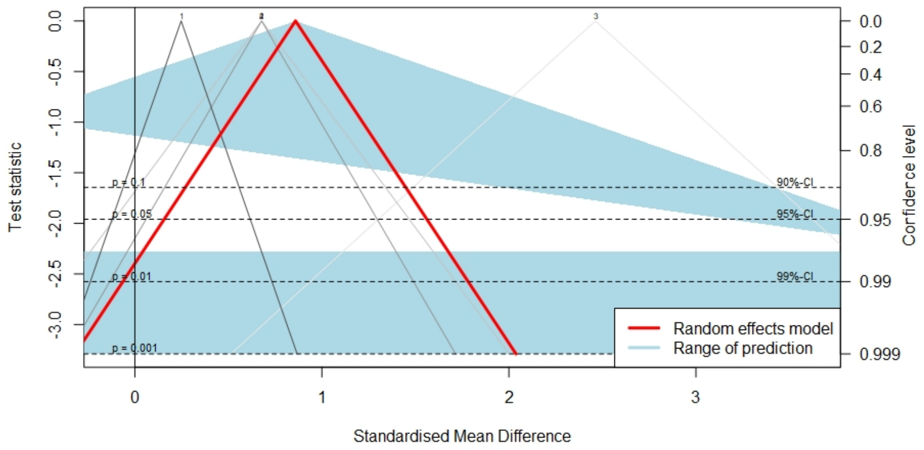

Leave-one-out analysis

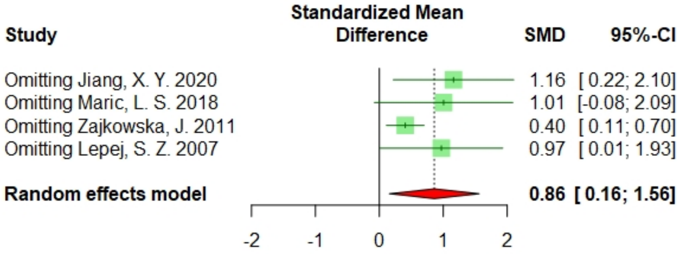

CXCL10 serum

Funnel plot

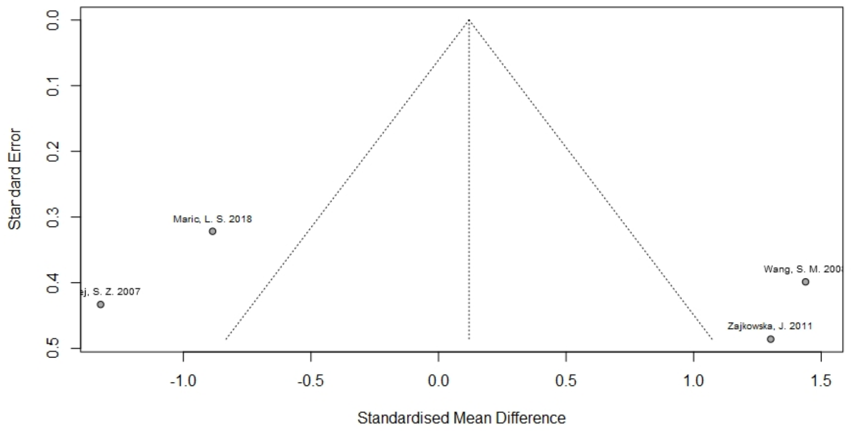

Drapery plot

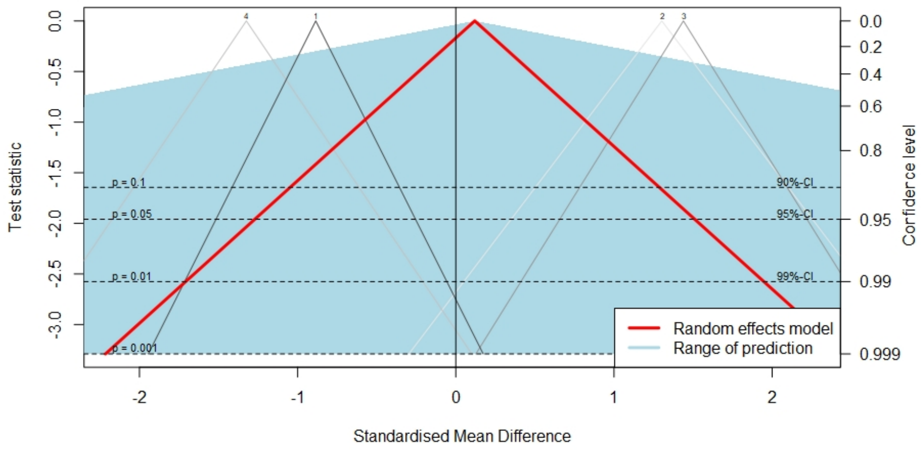

Leave-one-out analysis

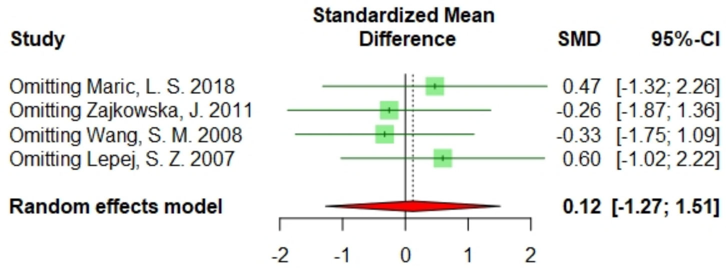

CXCL13 CSF

Funnel plot

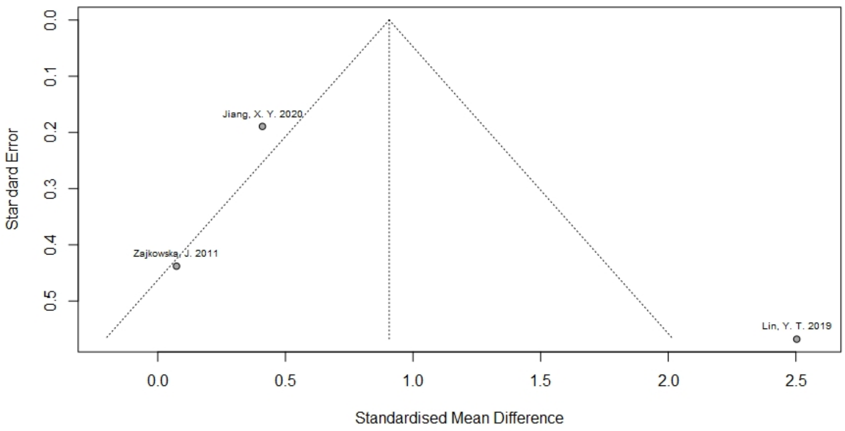

Drapery plot

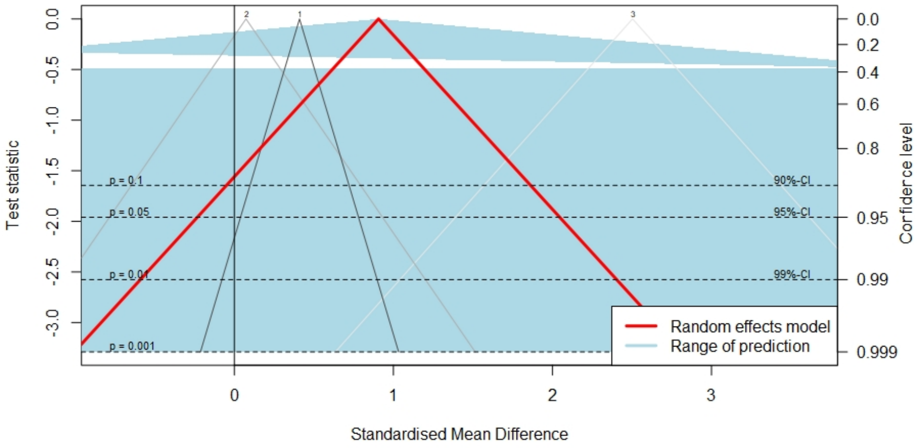

Leave-one-out analysis

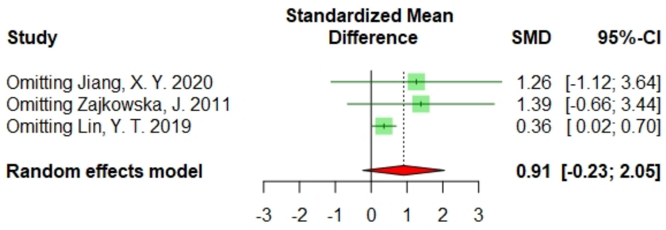

CXCL13 serum

Funnel plot

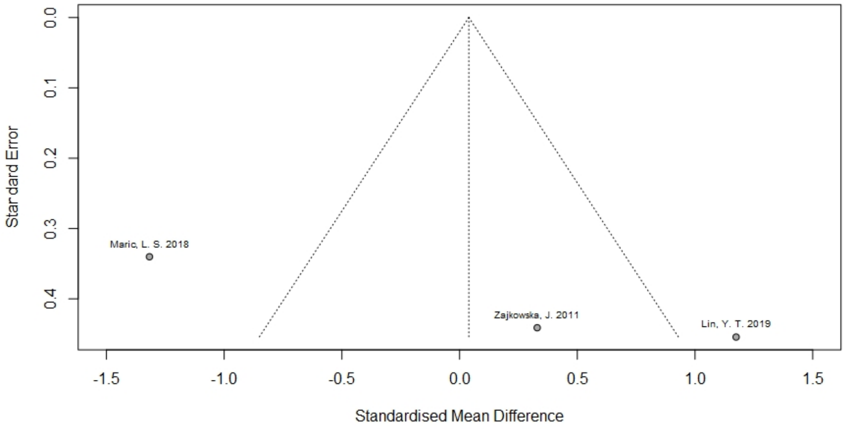

Drapery plot

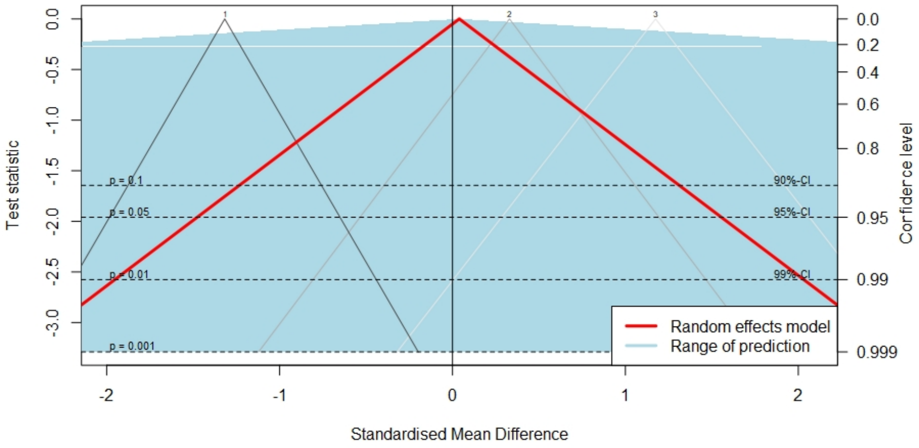

Leave-one-out analysis

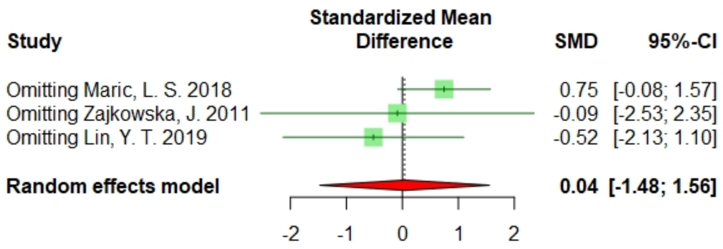

TNF-α CSF

Funnel plot

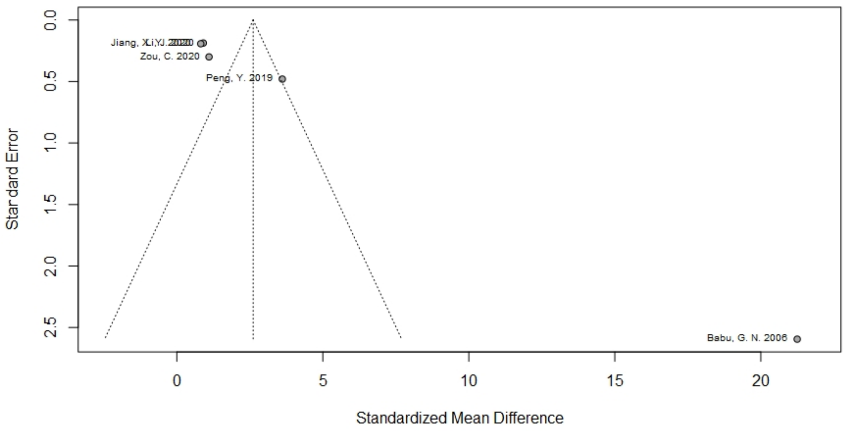

Drapery plot

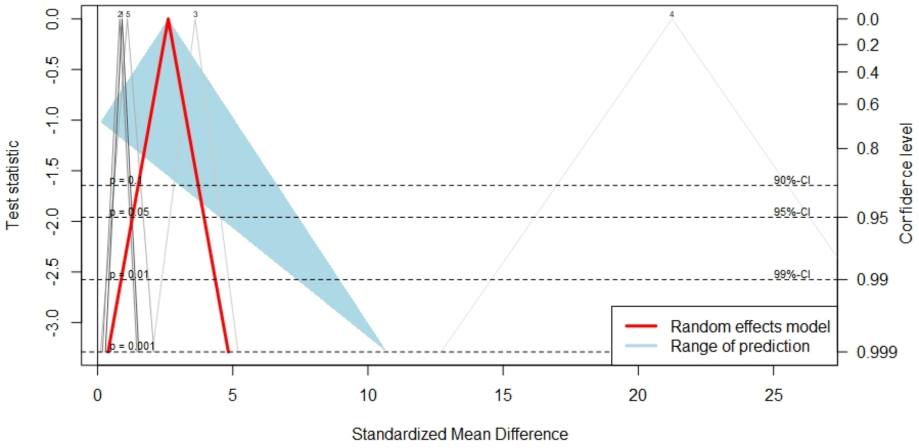

Leave-one-out analysis

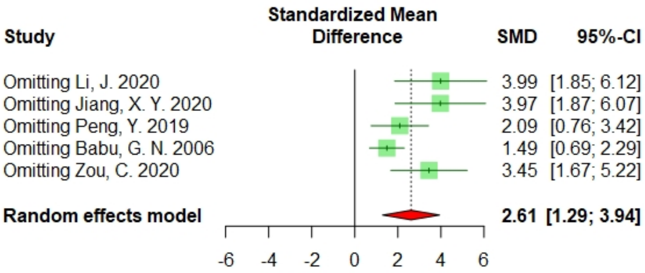

TNF-α serum

Funnel plot

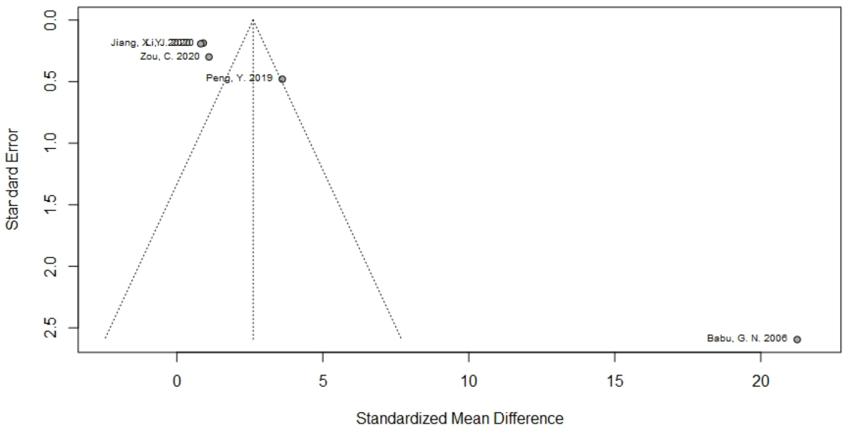

Drapery plot

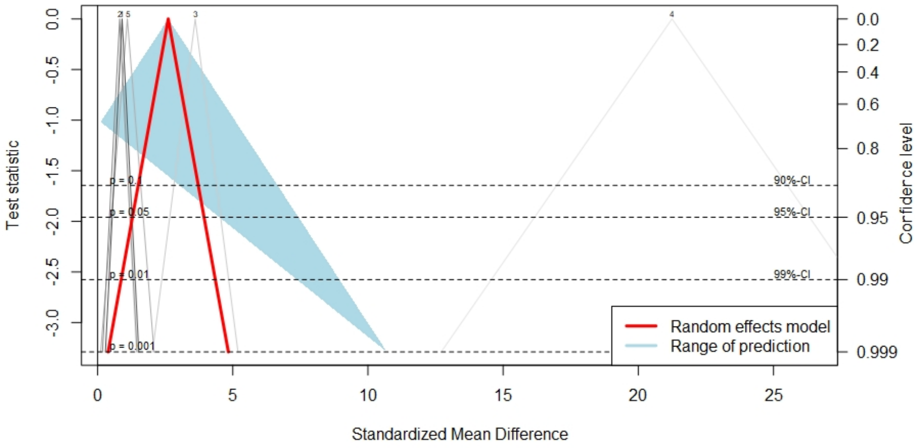

Leave-one-out analysis

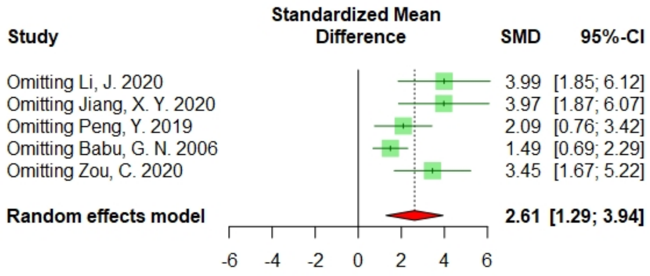

IFN-γ

Funnel plot

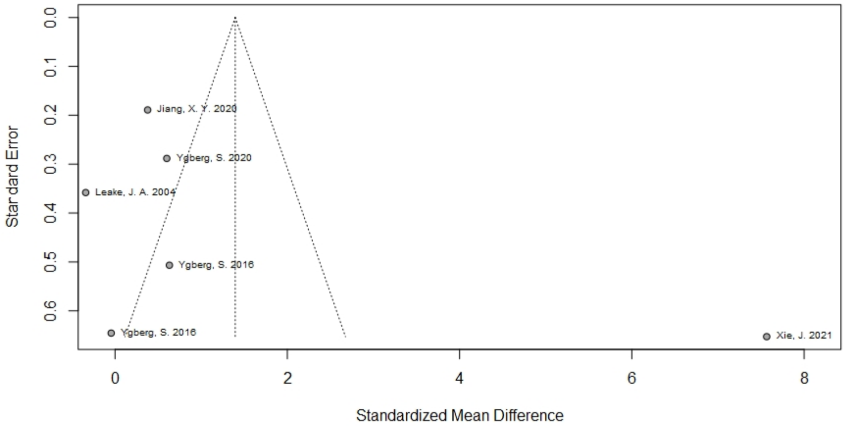

Drapery plot

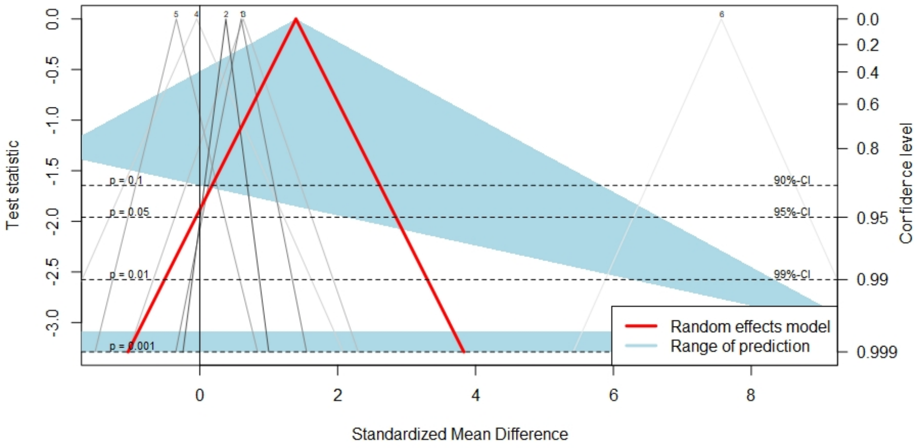

Leave-one-out analysis

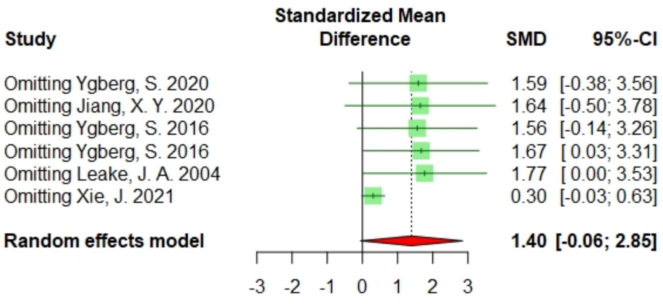

Supplement: S1 File — (PDF) [file pone.0273920.s004.pdf]
