# Supplementary figures and images for: Cytokines and chemokines profile in encephalitis patients: A meta-analysis
Source: PLoS One. 2022 Sep 1;17(9):e0273920. doi: 10.1371/journal.pone.0273920 (PMC9436077; doi:10.1371/journal.pone.0273920)

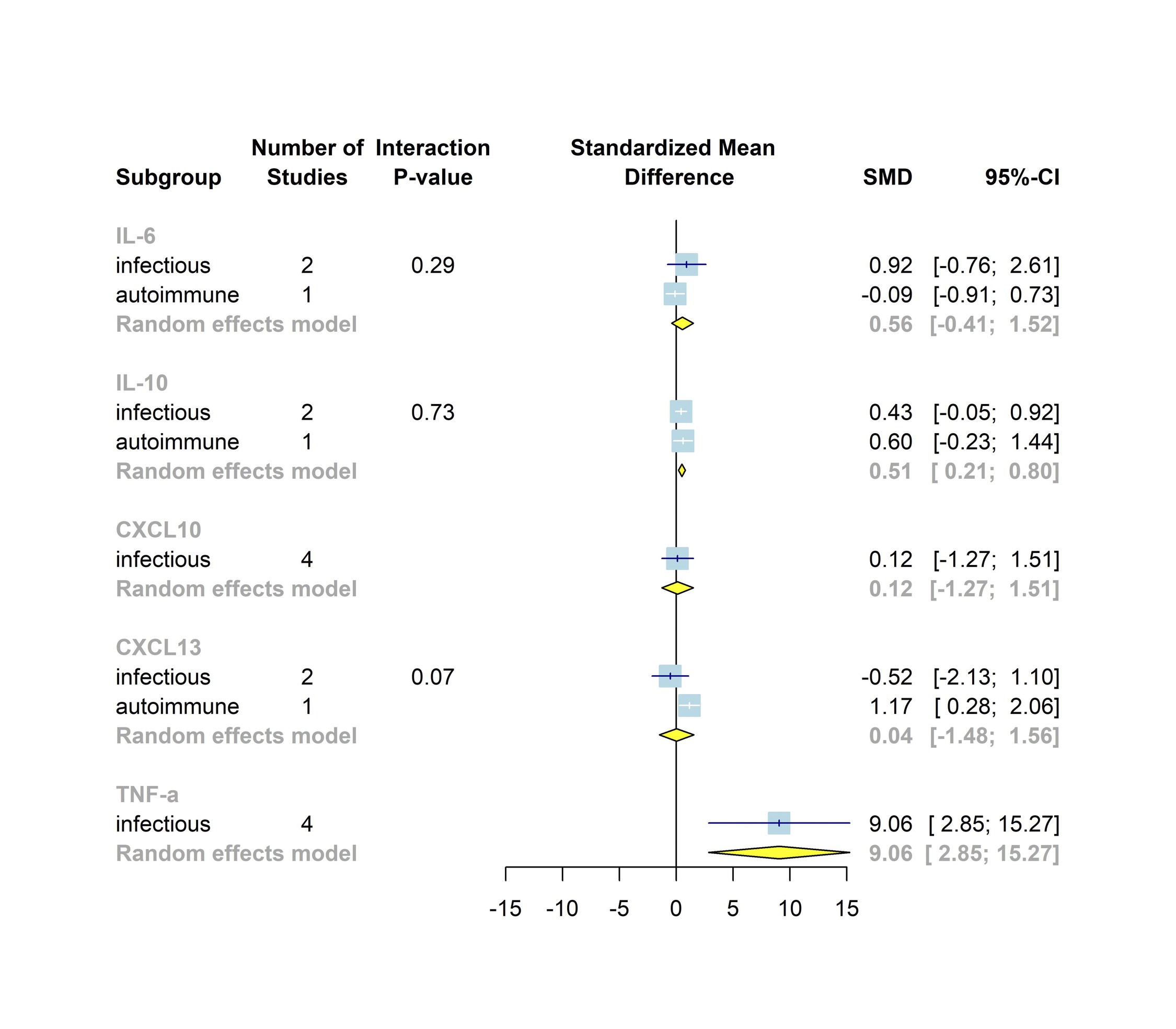

Supplement: S1 Fig — (TIF) [file pone.0273920.s005.tif]

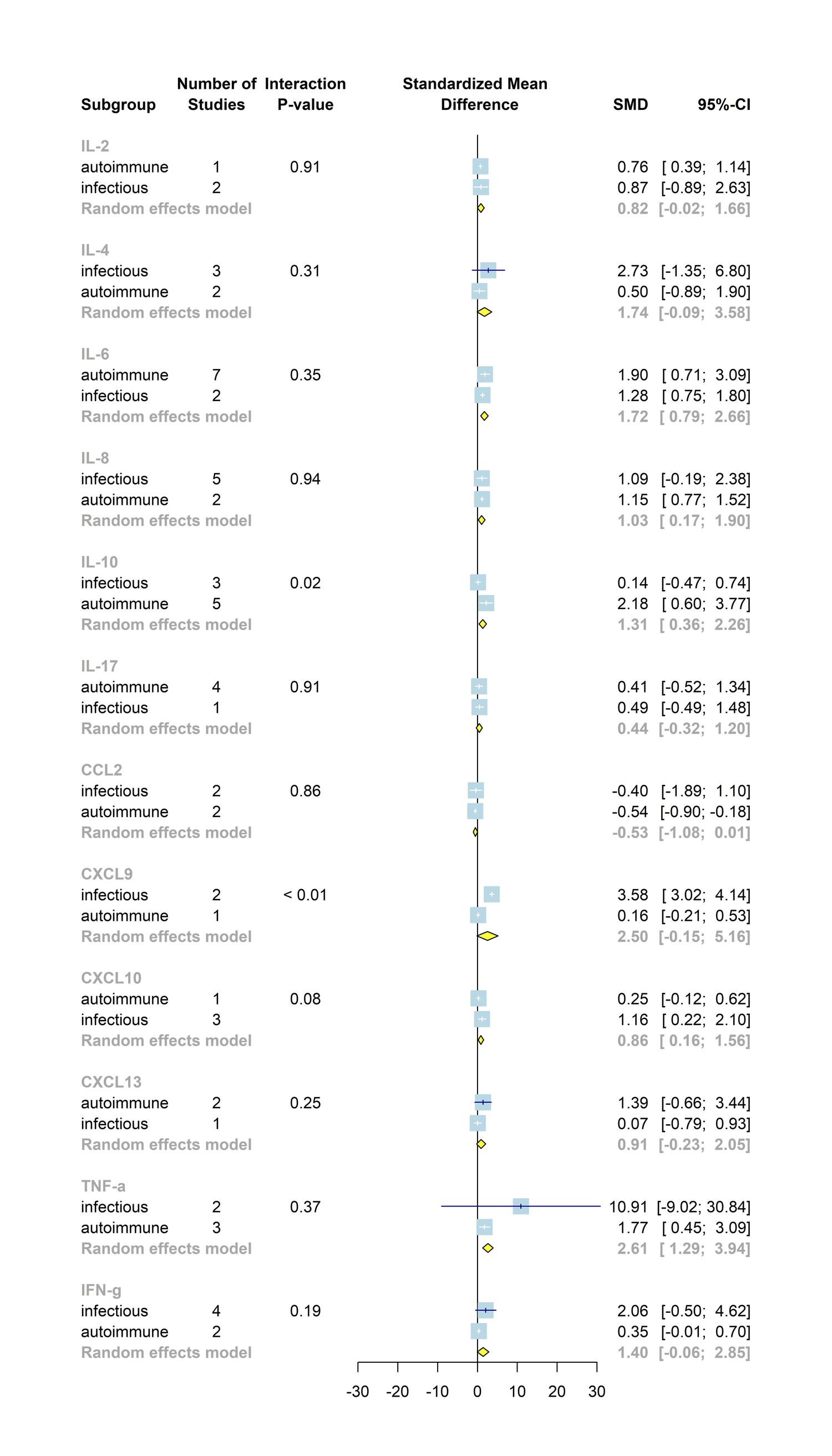

Supplement: S2 Fig — (TIF) [file pone.0273920.s006.tif]
